# Radiolabeling and quality control of therapeutic radiopharmaceuticals: optimization, clinical implementation and comparison of radio-TLC/HPLC analysis, demonstrated by [^177^Lu]Lu-PSMA

**DOI:** 10.1186/s41181-022-00181-0

**Published:** 2022-11-04

**Authors:** Eline L. Hooijman, Carolline M. Ntihabose, Thom G. A. Reuvers, Julie Nonnekens, Else A. Aalbersberg, Jordy R. J. P. van de Merbel, Judith E. Huijmans, Stijn L. W. Koolen, Jeroen J. M. A. Hendrikx, Erik de Blois

**Affiliations:** 1 Department of Radiology and Nuclear Medicine, Erasmus MC, 3015 CN Rotterdam, The Netherlands; 2 Department of Hospital Pharmacy, Erasmus MC, 3015 CN Rotterdam, The Netherlands; 3 Department of Molecular Genetics, Erasmus MC, 3015 CN Rotterdam, The Netherlands; 4 Department of Nuclear Medicine, The Netherlands Cancer Institute (NKI-AVL), Plesmanlaan 121, 1066 CX Amsterdam, The Netherlands; 5 Apotheek A15, Buys Ballotstraat 2, 4207 HT Gorinchem, The Netherlands; 6 Department of Medical Oncology, Erasmus MC Cancer Institute, Rotterdam, The Netherlands; 7 Department of Pharmacy and Pharmacology, The Netherlands Cancer Institute (NKI-AVL), Plesmanlaan 121, 1066 CX Amsterdam, The Netherlands

**Keywords:** Radiopharmaceutical production, Quality control, Radio-TLC analysis, HPLC analysis, [^177^Lu]Lu-PSMA, Radiolysis

## Abstract

**Background:**

Radiopharmaceuticals are considered as regular medicinal products and therefore the same regulations as for non-radioactive medicinal products apply. However, specific aspects should be considered due to the radiochemical properties. Radiopharmaceutical dedicated monographs are developed in the European Pharmacopoeia to address this. Currently, different quality control methods for non-registered radiopharmaceuticals are utilized, often focusing on radio-TLC only, which has its limitations. When the radiochemical yield (RCY) is measured by radio-TLC analysis, degradation products caused by radiolysis are frequently not detected. In contrast, HPLC analysis defines the radiochemical purity (RCP), allowing for detection of peak formation related to radiolysis. During the introduction and optimization phase of therapeutic radiopharmaceuticals, significant percentages of impurities, like radiolysed construct formation, may have consequential impact on patient treatment. Since more hospitals and institutes are offering radiopharmaceutical therapies, such as [^177^Lu]Lu-PSMA with an in-house production, the demand for adequate quality control is increasing. Here we show the optimization and implementation of a therapeutic radiopharmaceutical, including the comparison of ITLC and HPLC quality control.

**Results:**

Downscaled conditions (74 MBq/μg) were in concordance to clinical conditions (18 GBq/250 µg, 5 mL syringe/100 mL flacon); all results were consistent with an > 98% RCY (radio-TLC) and stability of > 95% RCP (HPLC). Radio-TLC did not identify radiolysis peaks, while clear identification was performed by HPLC analysis. Decreasing the RCP with 50%, reduced the cell-binding capacity with 27%.

**Conclusion:**

This research underlines the importance of the radiolabeling and optimization including clinical implementation and clarifies the need for cross-validation of the RCY and RCP for quality control measurements. Only HPLC analysis is suitable for identification of radiolysis. Here we have proven that radiolysed [^177^Lu]Lu-PSMA has less binding affinity and thus likely will influence treatment efficacy. HPLC analysis is therefore essential to include in at least the validation phase of radiopharmaceutical implementation to ensure clinical treatment quality.

## Background

Radiopharmaceuticals are considered as regular medicinal products and therefore be manufactured according to the principles and guidelines of the good manufacturing practices. Although much similarities between non-radioactive and radioactive medicinal products exist, the radioactive medicinal products have specific aspects to consider due to regulations on radioactive substances and due to radiochemical properties (Lange et al. 2015). Quality standards of radiopharmaceuticals in Europe are provided by the monograph on radiopharmaceutical preparation of the European Pharmacopoeia (Ph. Eur.) (EDQM 2022). Additionally, the EU GMP Annex 3 guideline—Manufacture of Radiopharmaceuticals 2008, also applies (European Commission 2008). For in-house preparations, additional guidance is given in the Good Radio-Pharmacy Practice (GRPP) (Gillings et al. 2021) and guidelines of the European Association for Nuclear Medicine (EANM) (Gillings et al. 2020).

In the Ph. Eur. monograph on radiopharmaceutical preparations, specific tests for the quality control of radiopharmaceutical preparations are described, including analytical tests for the radiochemical yield (RCY) and radiochemical purity (RCP) (EDQM 2022). As Coenen et al. (Coenen et al. 2017) described previously, the RCY is defined as the ratio (%) between labeled and free isotope and/or DTPA-bound isotope (incorporation). Additionally, the RCP is defined (EDQM 2022) as “the ratio, expressed as a percentage, of the radioactivity of the radionuclide concerned which is present in the radiopharmaceutical preparation in the stated chemical form, to the total radioactivity of that radionuclide present in the radiopharmaceutical preparation”.

Changes in the RCY and RCP may be caused by impurities introduced during radionuclide production, labeling procedures or chemical changes during preparation and storage. Furthermore, presence of radioisotopes in an aqueous solution causes the ongoing formation of radicals, leading to degradation of the peptide, thereby influencing the RCP, and in lesser extent the RCY (de Blois et al. 2012). This radiolysis should be identified during QC analysis. Furthermore, limited information is available about in vitro and in vivo binding of the described radiolysed product to the target cells, which is of importance for guaranteeing the effectivity and toxicity for clinical care.

According to the Ph. Eur., any method of analytical separation may be used in the determination of RCP (EDQM 2022). This includes (but is not limited to) thin-layer chromatography (TLC) and liquid chromatography (LC), as long as the separation technique (or combination of techniques) fit the purpose.

For radiopharmaceuticals with a Ph. Eur. monograph, the relevant radiochemical impurities and a method for separation are described in the specific monograph. However, for radiopharmaceuticals without a monograph, one should investigate possible radiochemical impurities and select a proper separation technique for the determination of the RCP. For in house preparations, the guide for the elaboration of monographs on radiopharmaceutical preparations by the EDQM (EDQM 2022) and the EANM guideline on the validation of analytical methods for radiopharmaceuticals can be useful for method selection and validation (Gillings et al. 2020). In general, a well-designed HPLC method outperforms radio-TLC in chromatographic separation of radiochemical impurities (de Blois et al. 2012). Radio-TLC, on the other hand, has no recovery issues and is usually easy and fast to perform and therefore may be an attractive alternative (EDQM 2022; Orhon, et al. 2022; Wang et al. 2020).

As more and more academic and general hospitals in Europe are offering radiopharmaceutical therapies like [^177^Lu]Lu-PSMA therapy (Weineisen et al. 2015) from an in-house production, the demand for adequate quality control methods is increasing (Kratochwil et al. 2019; Sadaghiani et al. 2022; Kalmthout et al. 2020). Prostate Specific Membrane Antigen (PSMA) labelled with Lu-177 is used for the treatment of metastatic Castration Resistant Prostate Cancer (mCRPC), it’s use has increased after FDA approval of [^177^Lu]Lu-PSMA-617 (Pluvicto^®^).

This manuscript aims to evaluate the radiolabeling optimization, clinical implementation and clarification of the significance of RCY and RCP measurements for quality control (QC), which is demonstrated by the preparation of [^177^Lu]Lu-PSMA-I&T, as an example. Moreover, this manuscript addresses the implications of radiolysed peptide and influence on the binding to the target.

## Methods

### Materials and chemicals

All used chemicals were in accordance with Good Manufacturing Practice (GMP) and Ph. Eur. regulations, except for 2.5 M sodium acetate from Sigma Aldrich (Zwijndrecht, The Netherlands) which was only used for research purposes. The stock solutions for the clinical labelling are prepared in quartz coated vials from Curium (Petten, The Netherlands). A self-developed stock solution is prepared by the A15 pharmacy (Gorinchem, The Netherlands), further referred to as quencher kit, containing gentisic acid and ascorbate (Tables 3, 5). PSMA-I&T was purchased from PiChem via ATT Scintomics (Fürstenfeldbruck, Germany). Lu-177 (EndolucinBeta) was purchased from ITM Medical Isotopes GmbH (München, Germany), DTPA (4 mg/mL) and Ethanol (96%), Apotheek A15 (Gorinchem, The Netherlands). The downscaled labelings with a molar activity of 74 MBq/nmol were performed in 0.5 mL Microtube vials from Sarstedt (Etten-Leur, The Netherlands).

For the radio-TLC, three mobile phases were included to develop a separation method for [^177^Lu]Lu-PSMA-I&T; (1) Acetonitrile was prepared in MilliQ water at a 1:1 (v/v) ratio. (2) Methanol was prepared in MilliQ water at a 1:1 (v/v) ratio with Ammonium Acetate (1 M) all purchased form Merck (Darmstadt, Germany). (3) Sodium citrate was purchased from J.T. Baker (Philipsburg, MT, USA) and was prepared at a concentration of 0.1, 0.5 and 1 M at pH 5. Additionally for radio-TLC validation purposes solutions of [^177^Lu]Lu-DTPA and [^177^Lu]Lu-PSMA-I&T (both 1.67 GBq/mL) were prepared.

For each HPLC (QC) injection, 6 MBq/100 µL was injected and was prepared in a 300 µL polypropylene vial from waters (Etten-Leur, The Netherlands), diluted in LC-MS grade Chromasolv 25% ethanol from Sigma Aldrich (Zwijndrecht, The Netherlands).

### Systems and settings

Validation of the processes and qualification of the dose calibrator, radio-(I)TLC-scanner, HPGe-detector, gamma counter and HPLC is performed conform the regulations set up by the EANM and based upon the GMP guidelines. Additionally, standardized geometries are used.

A dose calibrator was used for activity measurements for both the clinical and the research labellings (VIK-202 and VDC 404 Comecer, Castel Bolognese, Italy).

The ITLC analysis was performed by a radio-TLC-scanner (bSCAN, Brightspec, Zelik, Belgium) including a NaI(Tl) (calibrated energy 208 keV) scintillator detector, 2.54 × 2.54 cm crystal size, and digital multichannel analyzer (MCA), and automated gamma counter 2480 Wizard-2 (Perkin Elmer, Waltham, MA, USA) was used for Lu-177 activity measurements (calibrated energy: 208 keV).

HPLC analyses were performed by an Alliance 2695XE HPLC (Waters Chromatography B.V., Etten-Leur, The Netherlands) including a PDA detector (W2298) with dedicated software (Empower 3) connected to a 1 inch NaI(TI) Scionic crystal (Bunnik, The Netherlands) connected to a Canberra Osprey multichannel analyzer and signal amplifier (Zellik, Belgium). The samples are injected onto a RP-18 column (LiChrospher^®^ 100 RP-18 endcapped (5 µm), Merck, Darmstadt, Germany). A HPLC method is developed using two solvents: (A) 95% 0,1% TFA in water with 5% 0,1% TFA in ACN, and (B) 95% 0,1% TFA in ACN with 5% 0,1% TFA in water. The gradient used: 0–2 min 100% solvent A, 2–10 min 20% A to 80% solvent B, 10–15 min 100% solvent B, 15–20 min 100% solvent A, with a flow of 1 mL/min.

### Downscaled radiolabeling

A standardized labelling was introduced for validation purposes. Due to high peptide content and thus low molar activity, the labelling was performed consistently over time, which is described in Table 1.

**Table 1:** Reaction conditions of standardized labelling for qualification of equipment

Reaction component	Amount/final concentration
*During labelling*	
[^177^Lu]LuCl_3_	74 MBq
PSMA-I&T	7.5 µg
Gentisic acid	5 mM
Ascorbic acid	5 mM
Sodium Acetate	0.025 nM
*Directly after labelling*	
Ethanol (96%)	10% (v/v)
DTPA	2 mM
Final volume (Adjusted with MilliQ)	100 µL

For optimization of the radiolabeling conditions for clinical purposes, a downscaled model is implemented, using concentrations which are identical to concentrations and molar activity for patient dose preparation (120 MBq/nmol).

The Zanger et al. (2019) described the conditions for Lu-PSMA-617, which are comparable to the method described by Privé et al. (2020), leading to condition A (Table 2). Both methods were based on the existing [^177^Lu]Lu-DOTATATE method (Breeman et al. 2016), which has been used in the Erasmus MC for many years.

**Table 2:** Downscaled reaction conditions, based upon literature

Reaction components	Condition A: amount/final concentration
*During labelling*	
[^177^Lu]LuCl_3_	74 MBq
PSMA-I&T (0.1 M acetic acid)	1 µg
Gentisic acid	2 mM
Ascorbic acid	7 mM
*Directly after labelling*	
DTPA	2.5 mM
Final volume	40 μL

For optimization of the labelling conditions for the clinical efficiency, a quencher kit is used in different concentrations (condition 1–5, Table 3). Condition 1 is similar to condition A, but a kit matrix was applied. Additionally, 10% (v/v) ethanol is added (condition 2). The concentration of quencher kit was increased (condition 3–4). Finally, the dilution step was excluded (condition 5). The pH for all conditions was 4–4.5.

**Table 3:** Downscaled reaction conditions, optimized for clinical use

Conditions	Dilution	Gentisic acid (mM)	Ascorbate (mM)	Ethanol % (v/v)
1	80 ×	1.0	4.0	–
2	70 ×	1.2	4.5	10
3	40 ×	2.0	7.9	10
4	15 ×	5.4	21.2	10
5*	–	9.0	35.4	10

*Final volume for condition 1–4 = 50 μL, condition 5 = 30 μL

### Clinical radiolabeling

The radiolabeling of PSMA-I&T with Lu-177 is performed manually in a class A glovebox. The radiolabeling for 2 patient doses is performed by addition of 410 µL quencher kit to 250 µg PSMA-I&T. The quencher kit now containing PSMA-I&T is added to the [^177^Lu]LuCl_3_ (10 mL quartz coated vial) and incubated for 20 min at 90 °C in a dry heating block form Grant Instruments (Cambridge, UK) and cooled for 5 min afterwards. 1.12 mL DTPA, 1.17 mL ethanol, 0.95 mL quencher kit solution and 4 mL saline solution (0.9% NaCl) are added to the reaction vial, respectively. After displacement of the labelling solution into a vacuum vial, 2 mL saline (0.9% NaCl) is added, and 0.1 mL of the labelling solution is taken for quality control measurements. 4.1 mL of the labelling solution is added into a 5 mL syringe or 100 mL of saline (0.9% NaCl) to obtain the final patient solution. Additionally, the clinical labelling has also been performed with 34 GBq (4 patients), thereby increasing the Lu-177, PSMA-I&T (500 μg) and quencher kit (820 µL) in the labelling vial, and DTPA (2.24 mL), quencher kit (1.9 mL), ethanol (2.34 mL) and saline (8 mL) directly after labelling.

### Quality control

For patient release, different release criteria are set regarding the final activity, RCY and RCP. Intermediate activity measurements during- and after labelling are performed using the dose calibrator with validated geometries. To determine the RCY, radio-(I)TLC is used. An ITLC-SG strip [0.5 × 10 cm, dried 20 min at 160 °C from Agilent Technologies (Folsom, CA, United States) is spotted with 3 µL labelling solution, at 1 cm from the bottom of the strip and is dried for 30 s before placement in 250 µL 1 M Sodium-Citrate solution (pH 5). After separation, the strip is dried for 10 min, thereafter sealed in parafilm. For measurements, standardized settings for the drop point, endpoint, scanning time and regions of interest (ROI) for the ITLC-SG strip are used. The ITLC strip is cut at a retention factor (RF) of 0.7, each part is measured in the gamma counter. The pH is determined using pH paper from Merck (Darmstadt, Germany). An HPLC is prepared with and activity of 6 MBq/100 µL and was injected at time points t = 0 (directly after labelling), 4, 24 and 48 h, analysis is performed according to the earlier described settings.

### Radiolysis experiments PSMA-positive cell line

#### Radiolabeling for radiolysis formation

Radiolysis formation was set up by adapting the standardized labelling as described (*Table **1*), thereby using the quencher kit in different final concentrations to obtain a decreased RCP of [^177^Lu]Lu-PSMA-I&T. The final RCP’s applied to cells were 97%, 90%, 70% and 50% (HPLC confirmed). Conditions were stabilized after labelling by addition of an excess of quencher kit and saline to a final volume of 60 µL. Solutions for the cell experiments were prepared by dilution into RPMI-1640 medium (Gibco) at 0.5 MBq/mL (10^–9^ M).

#### Cell-uptake assay

The PC3-PIP cells were a kind gift of prof. Anna Orlova, Uppsala University, Sweden. Cells were cultured in RPMI 1640, Glutamax medium, supplemented with 10% fetal calf serum (Gibco), penicillin (100 units/mL) (Gibco) and streptomycin (100 units/mL) were all form purchased from Gibco, and in presence of puromycin (10 µg/mL) (Invivogen) every other passage. Cells were cultured at 37 °C with 5% CO_2_. 50.000 cells were seeded per well in a 24-well plate. After 24 h, 0.2 MBq in 1 mL of every condition was added per well in triplicate and plates were incubated for 30 min, t = 1 or 2 h at 37 °C. As a negative control and for background measurements, cells were incubated with medium. After incubation, cells were washed twice with phosphate buffered saline (PBS; Gibco). Subsequently, lysates were prepared by incubation with 1 M NaOH for 10 min at room temperature, after which the wells were washed twice with PBS and the washed fractions were pooled with the lysates. Radioactivity was measured in the automatic gamma-counter. Uptake was determined as the fraction of counts of the total added dose.

## Results

### Radiolabeling

For all radiolabeling, the release criteria were taken into account in concordance with the final patient dose. Criteria were set at a RCY > 98% for radio-(i)TLC and gamma-counter, the RCP > 95% for HPLC.

#### Optimization downscaled labelling conditions

A standardized labelling (Table 1) was performed for qualification of the equipment and resulted in an RCP of 96.6% ± *4.4*, t = 0, (n = 6). For optimization of the radiolabeling in regards to RCY and RCP, a downscaled model is implemented, as previously described. The radiolabeling based upon literature (Table 2) resulted in an RCP of 97.1% at t = 0 and decreased to 72.1% at t = 24 h.

Furthermore, conditions were applied as described previously in Table 3. To obtain an amount of quencher kit that is suitable for scaling the process to clinical production, an addition of increased volumes of quencher kit were tested, and resulted in a RCP increase from 88.0% to 94.1% after 24 h (Table 4). To minimize handling steps during clinical preparation in a cleanroom environment, the dilution step was eliminated thereby not decreasing the stability (condition 5) and resulted in a RCP > 95% up to 6 h.

**Table 4:** RCP results of different labelling conditions (74 MBq) for optimization as measured by HPLC (after t = 0, 2, 4–6, and 24 h)

Conditions	RCP (%)			
	t0	t2	t4–6	t24
1	97.5		96.1	88.0
2	98.5		98.0	91.5
3	98.9	98.4		93.0
4	97.3	98.4	96.8	93.7
5	97.0	98.8	95.5	94.1

To assess the effect of increased radioactivity (same concentration of components) to a larger volume, a scale step to 740 MBq was performed. Moreover, to evaluate the stability of the [^177^Lu]Lu-PSMA-I&T in saline for patient administration, the 740 MBq conditions were further diluted (Table 5). The dilution step includes saline (0.9% NaCl), ethanol (10% v/v of final volume) and quencher kit in the same concentration as during labelling (final volume 150 µL).

**Table 5:** RCP results for scaled labelling condition (740 MBq, after t = 0, 4 and 24 h)

Condition	Gentisic acid (mM)	Ascorbate (mM)	Ethanol % (v/v)	RCP (%)		
				t0 *(n* = *3)*	t4 *(n* = *2)*	t24 *(n* = *2)*
6	9.0	35.4	10	98.7 ± 0.4	97.4 ± 0.8	94.1 ± 1.2

#### From a downscale model to clinical production

The downscaled condition (condition 6) is scaled to clinical conditions. Subsequently, no significant difference (Table 6) can be observed between a labelling with 74 MBq (downscaled), 740 MBq and 18/32 GBq (clinical production), RCY was > 98% for all measurements.

**Table 6:** Comparison downscaled conditions to clinical conditions (t = 0 h)

	RCP (t = 0, %)
74 MBq	96.2 ± 1.0 (n = 3)
740 MBq	98.7 ± 0.5 (n = 3)
18 GBq	97.3 ± 0.8 (n = 3)
34 GBq	97.1 ± 0.5 (n = 3)

### Clinical radiolabeling

The labelling of 7.4 GBq/100 μg PSMA-I&T was dispensed in saline in either a syringe (5 mL) or a flask (100 mL). Table 7 summarizes the results of the RCY and RCP measurements. It can be noted that the RCY measured with the radio-TLC is > 99.3% for all time points, which is within the preset release criteria of RCY > 98%. With the gamma counter, a RCY > 98.6% for all time points was observed, which is also within the release criteria.

**Table 7:** RCY and RCP Production of clinical batches (after t = 0, 4, 24 and 48 h)

RCY (%), Radio-TLC-scanner				RCY (%), Gamma counter				RCP (%), HPLC			
t0	t4	t24	t48	t0	t4	t24	t48	t0	t4	t24	t48
*Syringe*											
> 99.9	> 99.9	> 99.9	> 99.9	> 99.9	> 99.9	99.8 ± 0.1	99.5	97.9 ± 0.1	97.5 ± 0.1	94.4 ± 0.1	90.8 ± 0.4
*Flask*											
99.5 ± 0.5	99.3 ± 0.5	99.7 ± 0.3	99.4 ± 0.6	98.6 ± 1.6	> 99.9	98.7 ± 1.3	99.3 ± 0.9	97.3 ± 0.7	96.5 ± 0.5*	92.8 ± 0.7	88.0 ± 2.7

Batches of the syringe (n = 2) or flask (n = 4), SD not displayed if measurements were all > 99.9%

*Performed n = 3

The RCP of the [^177^Lu]Lu-PSMA-I&T in the syringe (5 mL) was maintained > 97.5% up to 4 h, RCP in the flask (100 mL) was maintained > 96.5% up to 4 h (Table 7). Release criteria were set at an RCP of > 95%, administration to patients is therefore set to a maximum of 4 h after preparation.

### Cross-validation HPLC- and radio-TLC method

After qualification of the QC systems (dose calibrator, radio-TLC, gamma counter and HPLC) for the analysis of [^177^Lu]Lu-PSMA-I&T, the quality control is performed.

**Fig. 1:**
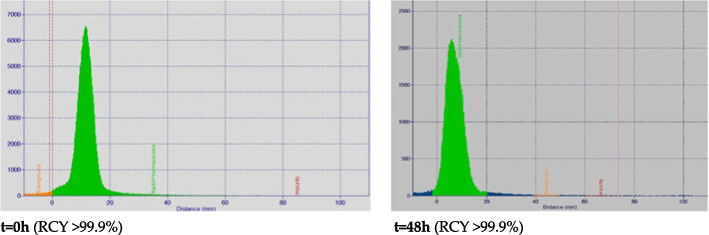
Radio-TLC at time point 0 and 48 h, for radiolysis evaluation

For the radio-TLC, of the three used mobile phases, only sodium citrate resulted in a reproducible separation of the different chemical forms (Lu-177, [^177^Lu]Lu-DTPA, [^177^Lu]Lu-PSMA-I&T) and no colloidal hydroxides were found in here described labelling procedure. The final radio-TLC parameters were: radiopharmaceutical (green, rf < 0–0.6), background (orange, rf < 0.6–0.7), and free and/or DTPA-bound Lu-177 (red, rf > 0.7–1.0), as illustrated by Fig. 1. It should be noted that the RCY for t = 0 and 48 h both shows to be > 99.9%, at t = 48 h, a different peak shape can be observed.

Results obtained from the radio-TLC-scanner were cross-validation with the gamma counter (Table 7). The RCY obtained by the gamma counter shows similar results, with an RCY of > 99.9% at t = 0 and RCY of > 99.0% at t = 48 h.

**Fig. 2:**
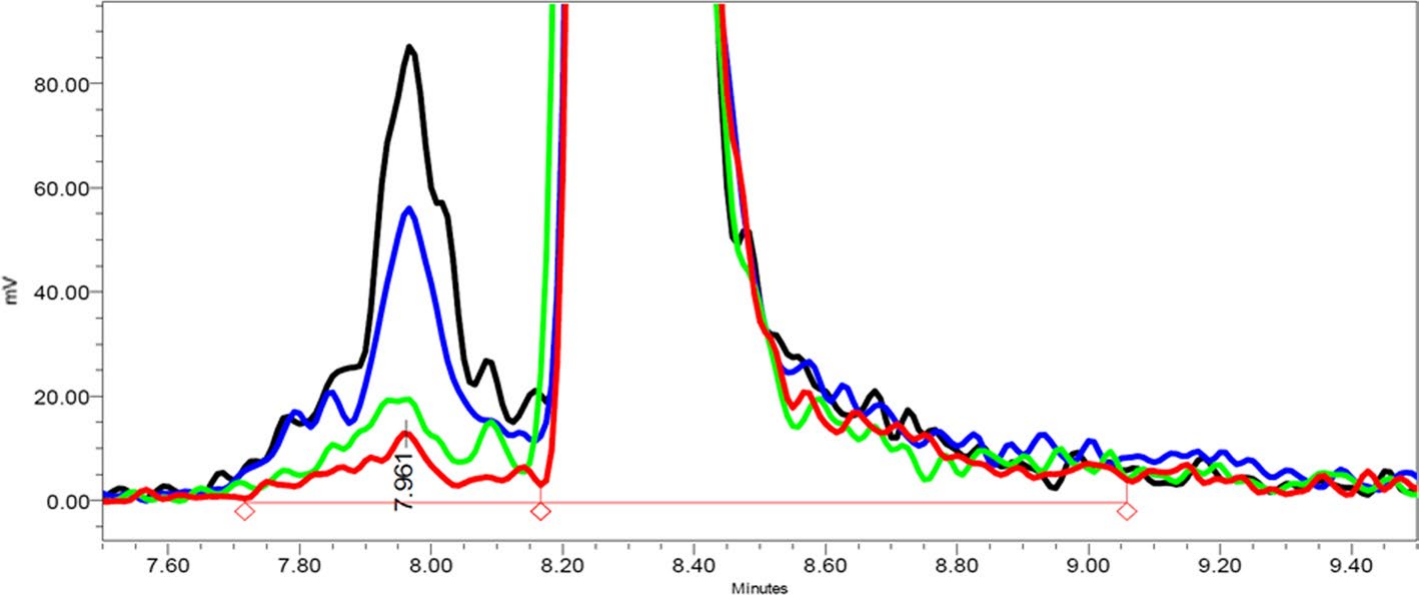
Increased radiolysis formation over time, as measured by HPLC analysis (t = 0 (red), t = 4 (green), t = 24 (blue) and t = 48 h (black))

Figure 2 shows the HPLC analysis of the RCP in time after labelling from 0 to 48 h. The main peak can be observed at a retention time (RT) of 8.35 min. In contrast to RCY obtained by TLC (Fig. 1), increased radiolysis formation was observed over time (RT 7.96 min).

### Impact of radiolysed [177Lu]Lu-PSMA-I&T

**Fig. 3:**
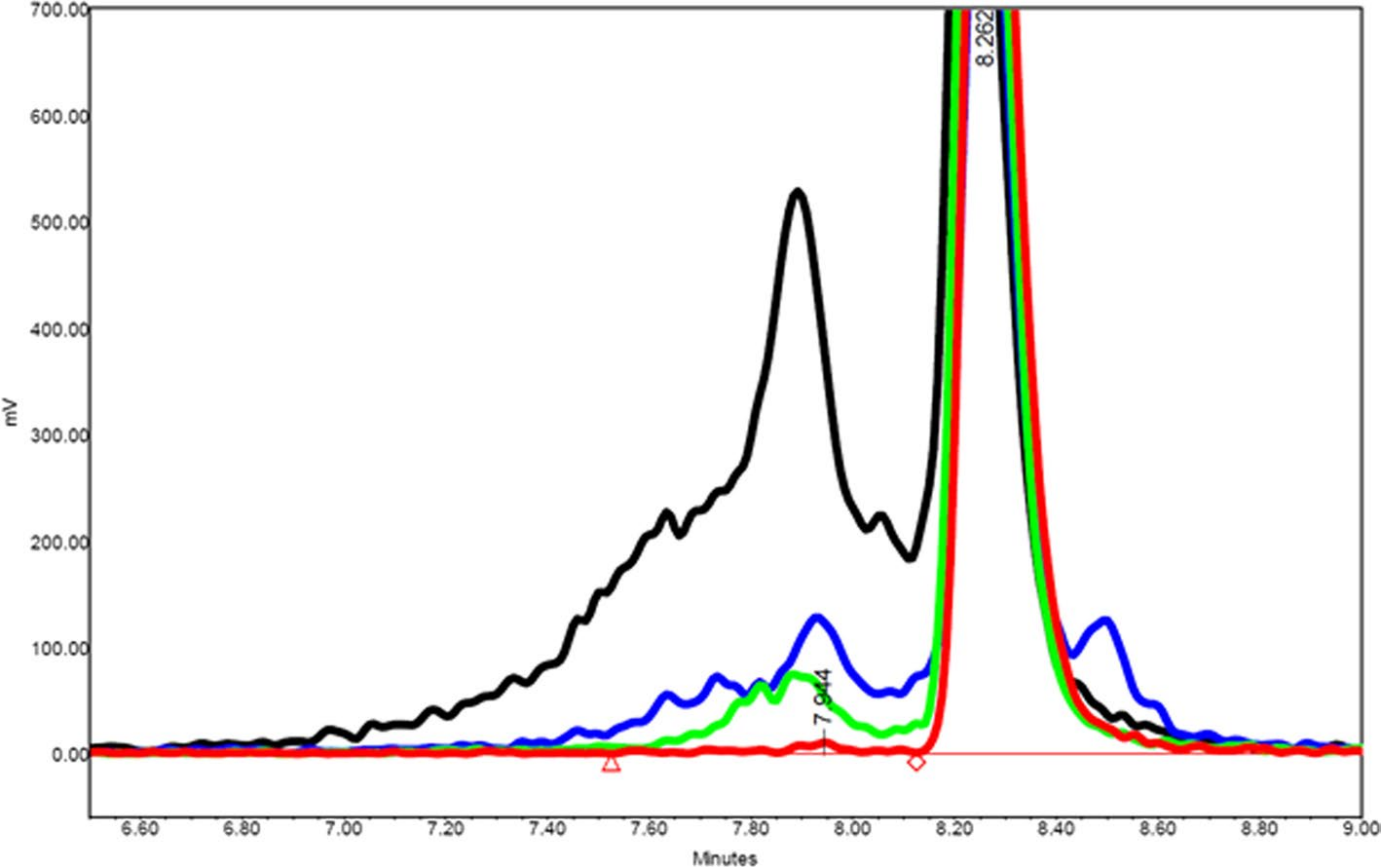
Radiolysed [^177^Lu]Lu-PSMA-I&T with a RCP varying from clinical labeling [97% (red)] and degraded radiopharmaceutical [90% (green), 70% (blue) and 50% (black)] for the cell-uptake assay

Controlled radiolysis was established, to evaluate the cell-binding with different RCP’s. Figure 3 shows the formation of radiolysis at a different RCPs; 97% (clinical conditions, 97.9 ± *0.4, n* = *3*), 90% (90.0 ± *1.8*, *n* = *3*), 70% (73.55 ± *7.6*, *n* = *3*) and 50% (53.1 ± *1.9*, *n* = *3*).

At a RCP of 50%, a 27.3% ± *3.5* (*n* = *3, t* = *0.5, 1 and 2 h*) decreased cell-uptake of was observed compared to a RCP of 97%. Consequently, a longer incubation time resulted in a higher cell-uptake, reaching a maximum after t = 2 h with 40% more uptake compared to t = 0.5 h (RCP of 97%).

## Discussion

This manuscript describes the evaluation of the quality control of radiopharmaceuticals for clinical implementation, in accordance with the Ph. Eur. monograph, EDQM, and EANM guidelines. Currently, different analytical methods are used for the quality control of produced Lu-177-labeled radiopharmaceuticals and are often limited to radio-TLC for release. This study highlights the importance of optimization before implementation of a radiopharmaceutical and the cross-validation of radio-TLC and HPLC analysis during the development of the quality control for the clinical implementation of (therapeutic) radiopharmaceuticals.

Different decisions were made to minimize the production time of the clinical radiolabeling. A quencher kit was introduced, thereby reducing the handling for the cleanroom personnel and subsequently, the radiation for personnel was also minimized. Optimization of the stability of the radiopharmaceutical is performed by using a downscale model thus reducing research costs, activity handling as well as the total waste. Due to presence of Lu-176 in carrier added produced Lu-177, and thus the lower specific activity, non-carrier added Lu-177 is required to obtain a high RCY and molar activity of 120 MBq/nmol.

As previously described (EDQM 2022; Gillings et al. 2020, 2021; Blois, et al. 2012), to be able to investigate possible radiochemical impurities a proper separation technique and method based upon determination of the RCY and RCP should be selected. Such a technique is dependent on the type of analytical technique (radio-TLC and HPLC), as well as the used method per technique. Worldwide, multiple methods are currently in use across different production facilities for non-registered radiopharmaceuticals. Due to the possibly high clinical impact of the impurities present in the administered final patient dose, impurity detection should be reviewed critically, and evaluated carefully in regards to the validation parameters according to the most recent EANM guidelines, so that all impurities are identified.

A frequently used analytical method is the radio-TLC scanner, which provides a practical and relatively quick option for measuring the labelled components within the labeling mixture. Since no recovery issues apply, and the possibility to identify colloidal hydroxides, radio-TLC analysis is recommended. During the validation phase of the radio-TLC-scanner for [^177^Lu]Lu-PSMA-I&T, some challenges were encountered. As an example, to obtain the most optimal peak separation and shape, different mobile phases needed to be tested. Additionally, use of any type of marking on the spot-location on the ITLC strip might interfere with the radiopharmaceutical separation. Furthermore, drying of the strip has shown to be essential; both drying the strip beforehand, as drying once the sample is spotted before sealing in parafilm to prevent inconsistent outcomes. As mentioned in the Ph. Eur., 1% of impurities should be detected.

When using the radio-TLC scanner, a balance should be found between scanning time and spotted activity. Additionally, a slight peak broadening was observed over time (Fig. 1), which indicates a change in radiopharmaceutical composition, and is likely caused by radiolysis formation which underlines the importance to perform a HPLC injection also.

The HPLC analysis can provide additional information on impurities, as the radiolysed peptide can be separated from the (non-)labelled product. During the validation phase, it needs to be emphasized that HPLC analysis with [^177^Lu]Lu-PSMA-I&T shows column related tailing, thereby underlining the importance of developing an accurate method.

Radicals in the aqueous solution react randomly with the radiopharmaceutical. If the pharmacophore maintains intact, the radiolysed product may still have affinity for the epitope and maintain its biological binding. Thereby, when assuming this specific radiolysed molecule as an impurity this might underestimate the total binding and thus the therapeutic effect. If the binding affinity of the specific radiolysed product remains, revision of the critical RCP parameter for patient release might be adapted accordingly. Likewise, identification of the structure of the radiolysis product, would be preferable to get more insight into the toxicity of the present impurities.

**Fig. 4:**
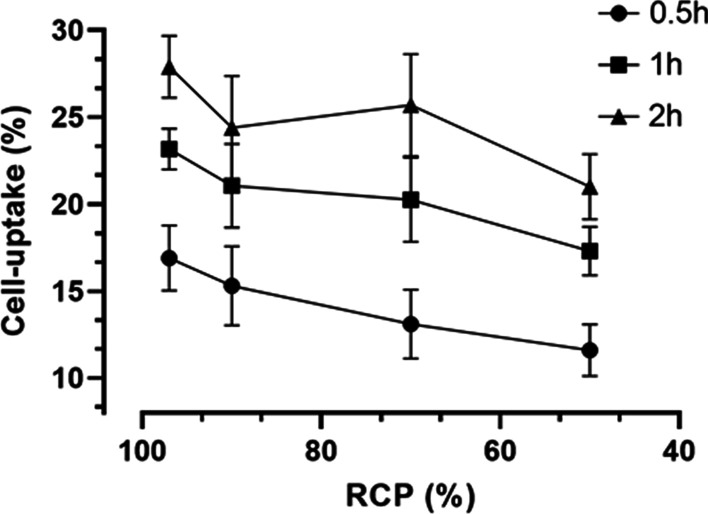
Correlation between the RCP (%, x-axis) and cell-uptake (%, y-axis), showing that a decrease in RCP leads to a decrease in cell-uptake (*n* = *3*)

Currently, to our knowledge, there is limited information on the binding properties of the formed radiolysed impurities, which are important to consider per radiopharmaceutical. Limited amounts of Lu-177 impurities (1% = 74 MBq) may already cause a side effect, as for example free Lu-177 has shown to accumulate in the skeleton (up to 60% of the Lu-177 radiopharmaceutical) and in the liver (up to 10%). With the use of DTPA the uptake in the bone and liver can be reduced and excretion via the kidneys is promoted (Sjögreen Gleisner et al. 2022). Limited information is available; however, it is expected that the possible impurities may cause toxicity and therefore, the binding properties of the impurities are investigated in a cell-uptake assay. A correlation can be observed as decreased RCP shows decreased cell-binding (in vitro, Fig. 4). In vivo data is required to evaluate the bioavailability of the radiolysed radiopharmaceutical to the tumor tissue.

This manuscript describes two different dispensing methods for patient administration. Firstly, the final patient dose was dispensed in a 5 mL syringe, thereby maintaining a higher RCP in time (~ 6 h). This creates flexibility and the possibility for shipment to other facilities. For in-house administration, preparation in a 100 mL saline flask was preferred due to the high osmolarity.

As shown in this research, the combination of both radio-TLC and HPLC analysis is of great importance. Nonetheless, after a sufficient dossier is built regarding stability data, the quality control frequency may be reduced. For further review of the [^177^Lu]Lu-PSMA-I&T QC analysis, an inter-institutional research setup would be beneficial to underline the robustness of the applied radiochemical and analytical analysis (Blois et al. 2019).

## Conclusion

This research shows the importance of the radiolabelling and optimization including the clinical implementation and the cross-validation of radio-TLC and HPLC analysis. We have proven that only by HPLC analysis it is possible to identify the radiolysed product. Additionally, the impact of radiolysis contributed to a reduced radiopharmaceutical binding to cells was shown. Both aspects could potentially influence clinical treatment quality and therefore patient safety.

## Data Availability

Please contact author for data request.
